# The Hepatitis C Cascade of Care for Opioid Agonist Therapy Recipients in Australia

**DOI:** 10.1093/cid/ciaf352

**Published:** 2025-07-09

**Authors:** Samara Griffin, Jason Asselin, Anna Lee Wilkinson, Michael W Traeger, Alisa Pedrana, Rebecca J Winter, Alexander J Thompson, Matthew Penn, Wayne Dimech, Basil Donovan, Margaret Hellard, Mark Stoové

**Affiliations:** Disease Elimination, Burnet Institute, Melbourne, Victoria, Australia; School of Public Health and Preventive Medicine, Monash University, Melbourne, Victoria, Australia; Department of Gastroenterology, St Vincent's Hospital, Melbourne, Victoria, Australia; Disease Elimination, Burnet Institute, Melbourne, Victoria, Australia; Disease Elimination, Burnet Institute, Melbourne, Victoria, Australia; School of Public Health and Preventive Medicine, Monash University, Melbourne, Victoria, Australia; School of Population and Global Health, University of Melbourne, Melbourne, Victoria, Australia; Disease Elimination, Burnet Institute, Melbourne, Victoria, Australia; School of Public Health and Preventive Medicine, Monash University, Melbourne, Victoria, Australia; Disease Elimination, Burnet Institute, Melbourne, Victoria, Australia; School of Public Health and Preventive Medicine, Monash University, Melbourne, Victoria, Australia; Disease Elimination, Burnet Institute, Melbourne, Victoria, Australia; School of Public Health and Preventive Medicine, Monash University, Melbourne, Victoria, Australia; Department of Gastroenterology, St Vincent's Hospital, Melbourne, Victoria, Australia; Department of Gastroenterology, St Vincent's Hospital, Melbourne, Victoria, Australia; Department of Medicine, University of Melbourne, Melbourne, Victoria, Australia; North Richmond Community Health, Melbourne, Victoria, Australia; National Serology Reference Laboratory, Melbourne, Victoria, Australia; Kirby Institute, University of New South Wales, Sydney, New South Wales, Australia; Disease Elimination, Burnet Institute, Melbourne, Victoria, Australia; School of Public Health and Preventive Medicine, Monash University, Melbourne, Victoria, Australia; Department of Medicine, University of Melbourne, Melbourne, Victoria, Australia; Department of Infectious Diseases, The Alfred and Monash University, Melbourne, Victoria, Australia; Disease Elimination, Burnet Institute, Melbourne, Victoria, Australia; School of Public Health and Preventive Medicine, Monash University, Melbourne, Victoria, Australia; Australian Research Centre in Sex, Health and Society, La Trobe University, Melbourne, Victoria, Australia

**Keywords:** hepatitis C virus, opioid agonist therapy, cascade of care, primary care, blood-borne viruses

## Abstract

**Background:**

People prescribed opioid agonist therapy (OAT) are a key population for hepatitis C virus (HCV) elimination. Health service engagement associated with OAT provision may facilitate hepatitis C testing and treatment. We aimed to quantify the HCV care cascade among people receiving OAT in Australia.

**Methods:**

We extracted linked data from individuals attending any of 58 clinics participating in the ACCESS national sentinel surveillance network of primary care and sexual health clinics from 1 January 2016 to 31 December 2023. Outcomes included evidence of any HCV test (antibody or RNA) or direct-acting antiviral (DAA) prescription at an ACCESS clinic after their first OAT prescription. RNA positive individuals were inferred antibody positive; individuals with a DAA prescription were inferred RNA and antibody positive. We determined the number of individuals at each stage of the following cascade by the end of the study period: (1) positive antibody, (2) positive RNA, and (3) DAA prescription.

**Results:**

Among 15 382 individuals prescribed OAT, 44% (6817) had an HCV antibody or RNA test after their first OAT prescription. Of these, 64% (4368/6817) were antibody positive by the end of the study period. Of these, 67% (2911/4368) were RNA positive, and of those, 69% (2007/2911) were prescribed DAAs.

**Conclusions:**

A high proportion of people prescribed OAT were not engaged in care by their OAT provider or across ACCESS network clinics, but when diagnosed, rates of treatment were high. Given high HCV antibody and RNA prevalence, integrating HCV care into regular OAT care should be a priority for HCV elimination in Australia.

## Teaser

Despite high hepatitis C antibody and RNA positivity among people on opioid agonist therapy (OAT) in Australia, only 44% had a hepatitis C test after their OAT prescription, and 69% of those eligible for treatment were prescribed direct-acting antiviral treatment.

The emergence of direct-acting antivirals (DAAs) led the World Health Organization (WHO) to set goals to eliminate hepatitis C as a public health threat by 2030 [1]. In Australia, DAAs were made publicly available and subsidized by the federal government from 2016, including in primary care settings. Since the availability of subsidized DAAs, >100 000 people have been treated for hepatitis C in Australia. However, in 2023, 5500 people were treated with DAAs, which is only 7% of the estimated 68 890 Australians with hepatitis C [2]. Despite significant investment in making DAAs widely available [3], if treatment rates do not improve, Australia is unlikely to reach elimination targets [4].

For Australia to meet WHO elimination goals, testing and treatment rates must increase, particularly in priority groups such as people who inject drugs [4, 5]. Strategies to increase testing and treatment rates among people who use drugs must focus on reducing barriers to care [1, 5], including competing daily priorities, experiences of stigma and discrimination, and complicated health systems [6–8]. Integrating hepatitis care into services that people who use drugs frequent can help minimize these barriers and increase engagement and retention in hepatitis C virus (HCV) care; service integration is a key component of current WHO global sector strategies for hepatitis C elimination [1]. Healthcare clinics providing opioid agonist therapy (OAT) are an ideal setting for hepatitis C care integration as they are a regular point of healthcare engagement for people who use drugs.

The diagnosis and treatment of hepatitis C typically requires multiple visits and loss to follow-up is common [9, 10]. Building on our previous analyses that showed low rates of hepatitis C antibody testing in the period following OAT commencement [11], we sought to understand the longer-term hepatitis C care outcomes and transition through each step of the cascade of care, from testing to diagnosis to treatment uptake, for individuals on OAT. Among individuals who received an OAT prescription within the DAA era, we aimed to quantify the proportion who progressed through each step of the hepatitis C cascade of care in a sentinel surveillance network of clinical services across Australia.

## METHODS

### Study Design and Setting

We conducted a retrospective cohort study. Data were from The Australian Collaboration for Coordinated Enhanced Sentinel Surveillance of sexually transmissible infections and blood-borne viruses (ACCESS). ACCESS is a national, sentinel surveillance network for monitoring, testing, treatment, and management of sexually transmissible infections (STIs) and blood-borne viruses (BBVs). Details of the ACCESS system are published elsewhere [12, 13]. ACCESS clinics are located in all Australian states and territories, and include general practice clinics, sexual health clinics, drug and alcohol services, community testing services, and hospital outpatient clinics. Clinics are invited to participate in ACCESS on the basis of providing services to and having high caseloads of people at risk of BBVs and STIs, including people who inject drugs. Clinics included in this analysis are a combination of government-funded sexual health and primary care centers and private practices that provide clinical services to people who inject drugs, including OAT prescription and/or hepatitis C testing and treatment.

### Data Source

Data from 58 primary care and sexual health clinics participating in ACCESS were included in this study. ACCESS data include electronic medical record data (including patient demographics, prescriptions, and pathology results) extracted using GRHANITE software. GRHANITE creates unique, nonidentifiable patient linkage keys, which allows linkage of patient records over time, within and across participating sites [13]. All data in this analysis were extracted using GRHANITE software. Of 58 clinics included in this analysis, all 58 sites performed HCV testing; 52 sites provide OAT services, and 44 sites provide hepatitis C treatment (DAA prescriptions). Data used for this analysis included OAT prescription drug and date of prescription, hepatitis C diagnostic test results (date of test, type of test, test result), DAA treatment prescription date, and individual's gender and age at the time of clinical consultation.

### Participants

This study included participants with at least 1 OAT prescription in the ACCESS database between 1 January 2016 and 31 December 2022. To limit our analysis to first treatments only while receiving OAT, individuals were excluded if they had an observed prescription for hepatitis C DAA treatment prior to the date of their first OAT prescription. We reviewed treatment episodes back to 2012.

Hepatitis C testing data were extracted through to 31 December 2023 to allow a minimum of 12 months of observation to receive hepatitis C care after first OAT prescription. Individuals were included once in analysis, that is, subsequent episodes of care for HCV infections or retreatments were not included. Individuals were observed from their first recorded OAT prescription during the study period until either 31 December 2023, or a recorded DAA prescription in the database. We report median observation time for individuals with and without a DAA prescription.

OAT prescription length in Australia is tailored to the individual's specific needs and can vary over time for individuals, based on dose stabilization and response to treatment. To estimate the regularity of OAT prescriptions, we calculated the number of yearly OAT prescriptions per individual and calculated the median and interquartile range (IQR). To estimate length of engagement in care, we calculated the median time between first observed and last observed OAT prescription. We report regularity of OAT prescriptions and length of engagement in care by stage of the HCV cascade of care (ie, not engaged in HCV testing or treatment, engaged in HCV testing, HCV RNA positive, and prescribed DAAs).

### Statistical Analysis

The primary outcome of this study was the number and proportion of individuals who received hepatitis C care. For this analysis, we have defined hepatitis C care as engaging in any testing or treatment related to hepatitis C (a record of receiving either an HCV antibody or HCV RNA test or DAA prescription). To explore timeliness of hepatitis C care, secondary outcomes included the time between stages of testing and treatment, and total time from first positive HCV antibody test to DAA prescription.

#### Hepatitis C Care Cascade

To estimate the hepatitis C testing and treatment cascade for people prescribed OAT, we included individuals with a record of any hepatitis C testing or treatment in the ACCESS database, after their first OAT prescription. Cascade stages were (1) positive HCV antibody test result, (2) HCV RNA test following HCV antibody positivity, (3) positive RNA test result, and (4) DAA prescription. To help account for missing data, we inferred previous stages for individuals based on subsequent known outcomes:

1. If individuals had an observed positive HCV RNA test but no positive HCV antibody test, they were inferred as HCV antibody positive.
2. If individuals had an observed DAA prescription but no positive test (HCV antibody or HCV RNA), they were inferred as HCV antibody and HCV RNA positive.

DAA prescription was measured by evidence of a recorded prescription in the electronic medical record data. Treatment uptake was calculated as the number of individuals with evidence of a DAA prescription divided by the number of individuals with an observed positive HCV RNA test.

#### Time Between Cascade Stages

To estimate the average time between stages of testing and treatment, we calculated the time (in days) for each of these 3 cascade steps:

1. A positive HCV antibody test and positive HCV RNA test.
2. A positive HCV antibody test and a DAA prescription.
3. A positive HCV RNA test and a DAA prescription.

#### Total Cascade Time

To estimate the total time in the cascade, a subgroup analysis was performed among patients who had all stages of the cascade observed, that is, a positive HCV antibody test, a positive HCV RNA test, and a DAA prescription (total time in the cascade).

Stages that were inferred were not included in these calculations. Time between events was determined for each individual, and the median time and IQR were calculated. To assess for differences by gender in hepatitis C care, median total time in the cascade was compared between males and females, with a rank-sum test for equality of medians (reported as a *P* value).

#### Timeliness of Treatment

To estimate the timeliness of treatment, we conducted a subanalysis for individuals who had an observed positive HCV RNA result and a DAA script. Because the study used surveillance data, comprised of electronic medical records, and observed “real world” hepatitis C journeys of patients, individuals have different observation periods and thus different periods of time to progress through the cascade. To compensate for this, we created a variable to compare timeliness of treatment. We categorized individuals into either (1) treated within 6 months of first testing HCV RNA positive, (2) treated over 6 months after first HCV RNA testing positive, or (3) not treated. We calculated the number and proportion of individuals treated within 6 months of first testing RNA positive by year of HCV RNA positivity. We explored timeliness of treatment by gender, with a rank test for equality of medians (reported as a *P* value).

### Ethics Approval

Ethics approval for ACCESS was provided by the human research ethics committees at Alfred Hospital (248/17), Central Australia (CA-19-3355), Northern Territory Department of Health and Menzies School of Health (08/47), University of Tasmania (H0016971), Aboriginal Health and Medical Research Council (1099/15), ACON (2015/14), Victorian AIDS Council/Thorne Harbour Health (VAC REP 15/003), and St Vincent's Hospital (08/051). As our study analyzes de-identified data collected under the auspices of public health surveillance, individual patient consent was not required. Individuals were able to opt out of the surveillance system if they wished.

## RESULTS

### Cohort Characteristics

A total of 15 514 individuals had at least 1 prescription for OAT at an ACCESS clinic between 1 January 2016 and 31 December 2022. Individuals with evidence of a DAA prescription (n = 132) prior to their OAT prescription were excluded. Of the 15 382 prescribed OAT with no prior DAA prescription, 10 471 (68%) were male and the mean age at first recorded OAT prescription during the study period was 42 years. Individuals attended 58 clinics across the surveillance network for both OAT prescriptions and HCV testing. All clinics contributed <15% of observations individually. The median observation time for individuals (n = 13 375) without a DAA prescription was 2152 days (IQR, 1257–2868 days). The median number of OAT prescriptions per year per individual was 10 (IQR, 7–13).

Of the 15 382 individuals with an OAT prescription, 8565 (56%) had no observed HCV care (test or DAA prescription) after their first recorded OAT prescription. Of individuals with no observed HCV care, the mean age at first recorded OAT prescription during the study period was 42 years, and 68% (n = 5852) were male. Of the 8565 individuals with no observed HCV care, the median number of yearly OAT prescriptions per individual was 9 (IQR, 6–12) and the median time in OAT care was 2302 days (IQR, 1641–2502 days).

#### Hepatitis C Care Cascade

**Figure 1.**
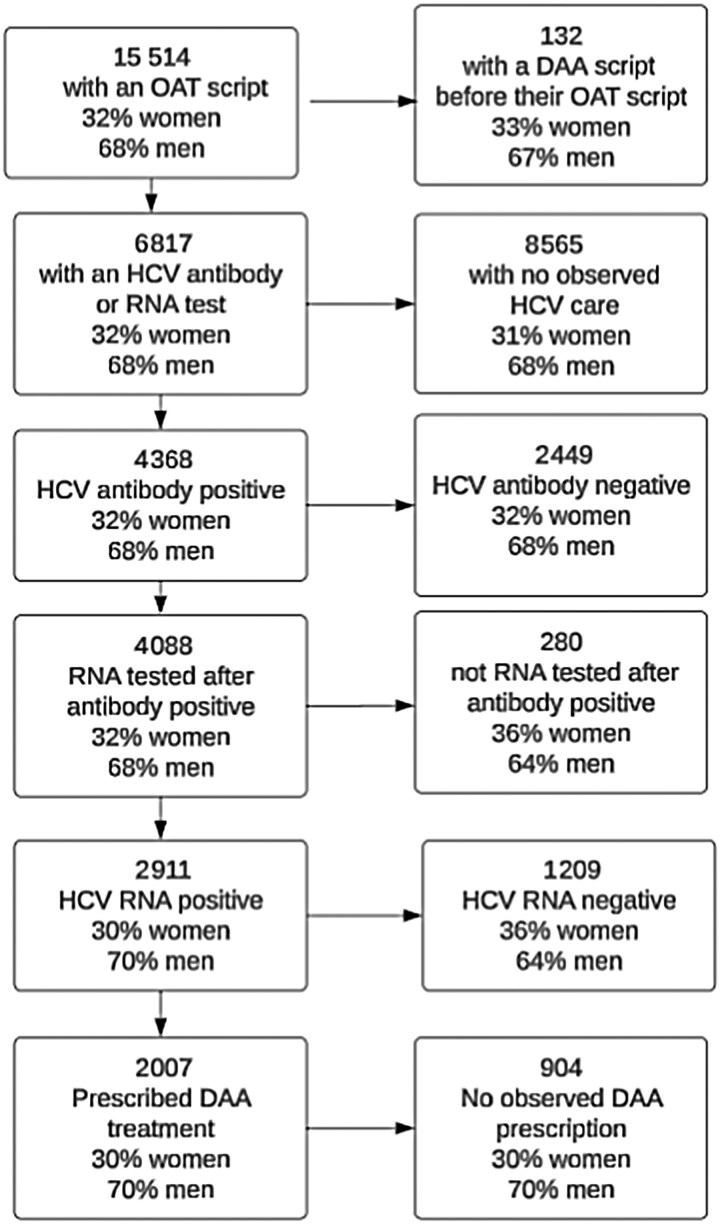
Patient flowchart of hepatitis C testing and treatment, Australian Collaboration for Coordinated Enhanced Sentinel Surveillance of sexually transmissible infections and blood-borne viruses (ACCESS), 2016–2023. Data include observed and inferred data. Individuals with reported gender other than male or female (n = 59) or missing gender were included in denominators but not reported in individual stages in figures due to small numbers, so some proportions of men and women may not add to 100%. Abbreviations: DAA, direct-acting antiviral; HCV, hepatitis C virus; OAT, opioid agonist therapy.

**Figure 2.**
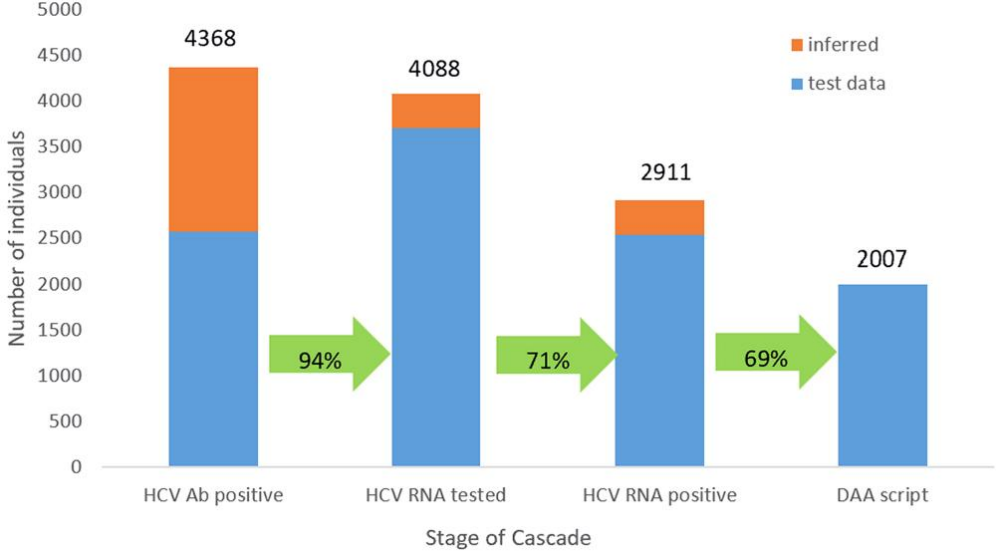
The hepatitis C cascade of care among individuals with at least 1 electronic prescription for opioid agonist therapy, Australian Collaboration for Coordinated Enhanced Sentinel Surveillance of sexually transmissible infections and blood-borne viruses (ACCESS), 2016–2023 (n = 4368). Abbreviations: Ab, antibody; DAA, direct-acting antiviral; HCV, hepatitis C virus.

Of the 15 382 individuals with an OAT prescription, 6817 (44%) had an HCV antibody or HCV RNA test (positive or negative) or DAA prescription recorded after their first recorded OAT prescription and were included in the cascade of care analysis. Of those tested or treated for HCV, the median age was 40 years and 4619 (68%) were male. The median number of OAT prescriptions per year per individual was 11 (IQR, 8–13) and the median time in OAT care was 1619 days (IQR, 813–2437 days). Of the 6817 individuals tested for HCV, 4368 (65%) were classified as HCV antibody positive by the end of the study period (2567 had a positive HCV antibody test record and 1801 were inferred antibody positive) (Table 1). Of 4368 antibody positive individuals, 4088 (94%) were classified as having an RNA test event by the end of the study period (3708 had an HCV RNA test recorded, and 370 were inferred RNA tested). Of the 4088 HCV RNA-tested individuals, 2911 (71%) were classified as HCV RNA positive by the end of the study period (2541 had a positive HCV RNA test recorded, and 370 were inferred RNA positive). Of the 2911 HCV RNA positive individuals, 2007 (69%) were prescribed DAAs by the end of the study period (Figures 1 and 2). The median observation time for individuals with a DAA prescription was 413 days (IQR, 175–773 days). Of those prescribed DAAs, the median number of yearly OAT prescriptions per individual was 13 (IQR, 8–13) and the median time in OAT care was 2433 days (IQR, 1830–2507 days). Of the 904 HCV RNA positive individuals without a DAA prescription, the median number of yearly OAT prescriptions was 10 (IQR, 7–13) and the median time in OAT care was 2318 days (IQR, 1643–2500 days).

**Table 1.** Population Breakdown Through Hepatitis C Testing and Treatment, ACCESS, Australia, 2016–2023 (N = 15 382)

Study Population	No. of Participants
Individuals on OAT with no prior DAA prescription	15 382
Individuals with no HCV engagement (ie, no recorded HCV antibody or RNA test and no DAA prescription	8565
HCV antibody-tested individuals	
Total	6817
Observed	3946
Inferred	2875
HCV antibody positive individuals	
Total	4368
Observed	2567
Inferred	1801
HCV RNA-tested individuals	
Total	4088
Observed	3708
Inferred	370
HCV RNA positive individuals	
Total	2911
Observed	2541
Inferred	370
Individuals with a DAA prescription	2007

Abbreviations: ACCESS, Australian Collaboration for Coordinated Enhanced Sentinel Surveillance of sexually transmissible infections and blood-borne viruses; DAA, direct-acting antiviral; HCV, hepatitis C virus; OAT, opioid agonist therapy.

#### Time Between Cascade Stages

Analyses of time between cascade stages included 4368 individuals who had an observed HCV test or DAA prescription. The time between first OAT prescription and first observed HCV antibody test was 327.5 days (IQR, 61–875 days). Of those with an observed positive HCV antibody test (n = 2567), the median time between first OAT prescription and first positive HCV antibody test was 414 days (IQR, 98–1026 days). Of those with observed positive HCV antibody and subsequent positive HCV RNA test (n = 1087), the median time between first positive HCV antibody test and first positive HCV RNA test was 0 days (IQR, 0–0; 90th percentile, 37 days; maximum, 2134 days). The median time between first positive HCV RNA test and DAA prescription (n = 1637) was 87 days (IQR, 32–254 days) (Table 2).

**Table 2.** Time Between Stages Analysis for Hepatitis C Testing and Treatment, ACCESS, Australia, 2016–2023

Stage	Median Time, Days (IQR)
First OAT prescription to first HCV antibody test (n = 3946)	328 (61–875)
First OAT prescription to first HCV antibody positive result (n = 2567)	414 (98–1026)
First OAT prescription to first HCV RNA positive result (n = 2541)	224 (70–532)
First OAT prescription to first HCV RNA test (n = 1309)	0 (0–0)
First HCV antibody positive result to first HCV RNA positive result (n = 1087)	0 (0–0)^a^
First HCV RNA positive result to first prescription for DAAs (n = 1637)	87 (32–254)

Abbreviations: ACCESS, Australian Collaboration for Coordinated Enhanced Sentinel Surveillance of sexually transmissible infections and blood-borne viruses; DAA, direct-acting antiviral; HCV, hepatitis C virus; IQR, interquartile range; OAT, opioid agonist therapy.

^a^90th percentile 37 days, maximum 2134 days.

#### Total Cascade Time

**Figure 3.**
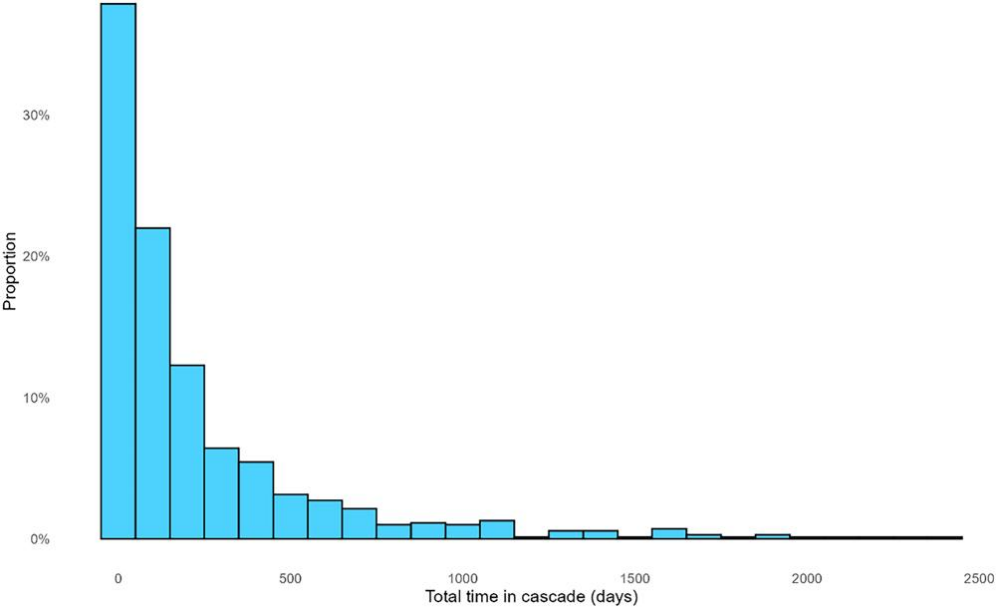
Total time in cascade from first hepatitis C virus antibody positive result to direct-acting antiviral prescription for individuals with at least 1 electronic prescription for opioid agonist therapy, Australian Collaboration for Coordinated Enhanced Sentinel Surveillance of sexually transmissible infections and blood-borne viruses (ACCESS), 2016–2023 (n = 723).

Subanalysis of total time in the cascade included 700 individuals who had all cascade stages directly observed. The median total time in the cascade for individuals who had all cascade stages observed was 92 days (IQR, 32–300 days). The distribution of total time in the cascade for all individuals is shown in Figure 3. The median total time in the cascade was longer for women (98 days [IQR, 40–286 days]) than for men (87 days [IQR, 30–313 days]) (*P* = .68).

#### Timeliness of Treatment

**Figure 4.**
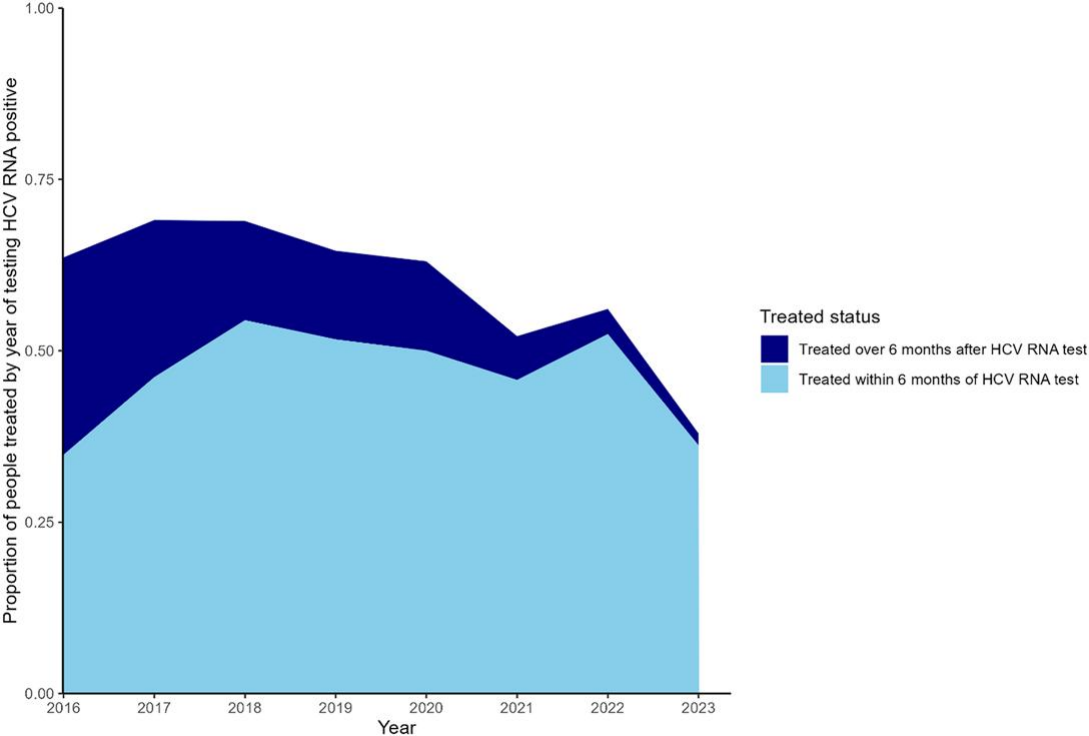
Proportion of people receiving a direct-acting antiviral prescription within 6 months of testing hepatitis C virus (HCV) RNA positive, by year of first testing HCV RNA positive, Australian Collaboration for Coordinated Enhanced Sentinel Surveillance of sexually transmissible infections and blood-borne viruses (ACCESS), 2016–2023 (n = 2541).

Among those who had an observed positive HCV RNA test and a prescription for DAAs, the overall proportion of individuals treated within 6 months of first testing RNA positive was 52% (1327/2541) and did not vary by gender (*P* = .72). The proportion of individuals treated within 6 months of first testing RNA positive fluctuated yearly from 35% (362/1039) in 2016 to a peak of 54% (177/325) in 2018 (Figure 4).

## DISCUSSION

In this sentinel surveillance network incorporating 44 primary care clinics, 13 sexual health clinics, and 1 hospital outpatient clinic, for 56% of people prescribed OAT there was no evidence that they were engaged in hepatitis C care by their OAT provider or across the network. This is a higher proportion of individuals engaged in care than self-reported Australian data among people who use drugs [14, 15]. Among the 44% of people who received hepatitis C testing, the average time between first OAT prescription and receiving an HCV antibody test was just under a year. Given the high HCV antibody (65%) and HCV RNA (71%) prevalence among those tested for hepatitis C, and DAA uptake of 69% among those HCV RNA positive, these data suggest significant missed opportunities to engage a group of people at high risk in hepatitis C care and treatment. Even when care was provided, treatment was not initiated quickly, with an average of approximately 3 months between being confirmed HCV RNA positive and being provided with a DAA prescription. Timely postdiagnosis treatment is important to prevent individual morbidity and mortality and reduce time spent viremic and potential onward transmission.

Our findings show that once diagnosed, retention in hepatitis C care is high among those prescribed OAT. Of those who were HCV antibody positive, nearly all were tested for HCV RNA (94%), likely due to clinicians requesting reflex HCV RNA testing (median time between positive HCV antibody test and RNA test, 0 days). Further, more than two-thirds of HCV RNA positive individuals were prescribed DAA treatment (69%). This is higher than a previous ACCESS study by Traeger et al looking at hepatitis C testing across all individuals in the ACCESS database (ie not restricted to those on OAT) [10], which found that 45% of HCV RNA positive individuals were prescribed DAAs. Traeger et al reported HCV RNA prevalence of 56% among those tested for HCV RNA. While this study and the study by Traeger et al would have population crossover (as both analyses used the ACCESS database), the difference in retention between these analyses highlights the potential for high-quality hepatitis care through OAT providers due to recurring engagement with patients [16].

As well as the high proportion of people receiving OAT in the service network who were never engaged in hepatitis C testing and care during the observation period, our results also show that almost 1 in 3 who were diagnosed with chronic HCV RNA infection did not have evidence of a DAA prescription. While some of these individuals may have been treated outside of the ACCESS network, these data indicate a lack of integrated and person-centered care and a significant health system failure, where opportunities to treat hepatitis C in OAT settings are not capitalized on. The provision of hepatitis C care in OAT settings has been shown to be highly acceptable, with clients citing benefits associated with convenience and established trust with service providers [17]. Despite the potential benefits to clients and system efficiencies derived from integrated care models, OAT providers face both practitioner and health system-level barriers to providing hepatitis C care to clients, including venipuncture requirements for testing, lack of awareness of testing and treatment guidelines, and funding issues [18]. Improved integration of drug treatment and hepatitis C models of care that capitalize on established relationships and regular clinical engagement in primary care settings has the potential to play a key role in Australia reaching WHO hepatitis C elimination goals. In Australia, examples of practice “toolkits” that were developed to better integrate hepatitis C care into general practice [19] and pharmacy settings [20] could be readily adapted to provide specific guidance for OAT prescribers.

In our analysis of time between stages, we found that the median time between first recorded OAT prescription and first recorded HCV antibody test was close to a year, despite Australian OAT treatment guidelines recommending screening for blood-borne viruses as a clinical priority [21]. This finding is in line with other studies that have shown a minority of people on OAT receive antibody testing within 12 months of their first recorded OAT prescription [11]. While a median of 9 OAT prescriptions per year indicate some level of retention in OAT care, engagement in OAT care is complex and can fluctuate for a multitude of reasons; any engagement in OAT care is an important opportunity to provide additional healthcare [22]. These findings highlight a need to develop client and practitioner-acceptable models of OAT that facilitate opportunities for hepatitis C care sooner after OAT initiation to capitalize on healthcare engagement. Our findings indicate that once engaged in testing, treatment is initiated quite quickly, with a median time of 87 days between first HCV RNA positive test and DAA prescription. Improving timeliness of treatment is an important measure to reduce time spent viremic [23]. Reducing time spent viremic not only limits onward transmission of hepatitis C, but at an individual and population level reduces hepatitis C-related morbidity such as liver failure and cirrhosis. In our study, the median time between the first HCV RNA positive test and DAA prescription was 3 months; there is clearly room for improvement here.

One limitation of this study is that episodes of care that occur outside the ACCESS network are not captured, which may underestimate the proportion of people who progressed through testing and treatment stages. This limitation is exacerbated by the fact that the ACCESS network does not include tertiary care services, which provided a considerable amount of DAA treatment during the first few years of DAA availability [4]. Further, our analysis only considered first DAA prescriptions observed within the ACCESS database. This was done to have consistency across the time-to-treat analysis, as individuals going through the cascade at subsequent times may progress more quickly due to experience with the process. We have excluded any observed retreatments through an ACCESS clinic; however, this does not exclude individuals who had their first treatment outside of ACCESS. While many models of hepatitis C care do not prioritize sustained virological response (SVR) testing due to the efficacy of DAAs, it is a limitation of this analysis that we do not report on SVR testing. ACCESS data do not have clinical indicators for HCV RNA testing, meaning that we would not be able to ascertain if RNA tests were conducted for cure or diagnosis purposes. Similarly, ACCESS treatment initiation data are restricted to DAA prescription; we do not know if individuals were dispensed or commenced this prescription. Furthermore, the primary analysis uses longitudinal retrospective surveillance data, meaning that individuals had different observation periods to determine progress through the cascade. However, our longitudinal surveillance was able to determine “real world” progress through the cascade of care that was not artificially constrained by fixed points of cohort censorship. Finally, we have not accounted for spontaneous clearance in our calculation of the population eligible for treatment and treatment uptake.

## CONCLUSIONS

Despite high prevalence of HCV infection among those receiving hepatitis C testing, a majority of people on OAT in ACCESS had no observed hepatitis C testing or treatment. Of those engaged in testing, access to hepatitis C diagnostic testing was delayed, and one-third of those who had a current hepatitis C infection did not receive treatment within the study period. Health system improvements are required to help service providers provide timely, quality hepatitis C care to this population. If Australia is to reach WHO elimination goals, better integrated and person-centered models of hepatitis C care will be required.

## Notes

***Author contributions*.** S. G.: Conceptualization, methodology, formal analysis, writing—original draft. J. A.: Conceptualization, methodology, supervision, data curation, project administration, validation, writing—review and editing. A. L. W.: Conceptualization, methodology, supervision, data curation, formal analysis, validation, writing—review and editing. M. W. T.: Methodology, validation, data curation, writing—review and editing. A. P.: Methodology, writing—review and editing. R. J. W. and A. J. T.: Supervision, writing—review and editing. M. P.: Resources, Writing—review and editing. W. D. and B. D.: Funding acquisition, writing—review and editing. M. H. and M. S.: Conceptualization, funding acquisition, supervision, writing—review and editing.

***Acknowledgments.*** The authors acknowledge the contribution of the ACCESS Team members who are not co-authors of this article, including: Allison Carter, Htein Linn Aung, Rebecca Guy, and Greta Baillie, Kirby Institute, University of New South Wales (UNSW) Sydney; Alexis Apostolellis, Australasian Society for HIV Medicine; Aaron Cogle, National Association of People with HIV Australia; Brendan Quinn, Victoria Department of Health; Clare Bradley, ATLAS; Daniel Coase, Federation of Ethnic Communities’ Councils of Australia; Dash Heath-Paynter, Health Equity Matters; David Lewis, Western Sydney Sexual Health Centre; David Nolan, Royal Perth Hospital; David Templeton, Royal Prince Alfred Hospital; Edward Huddy, Ministry of Health; Emma Sanguineti, Queensland Ministry of Health; Eric Chow and Kit Fairley, Melbourne Sexual Health Centre; Florin Douglas, Deakin University; Janaki Amin, NSW Ministry of Health; Jane Davies, Menzies School of Health Research; Jane Hocking, University of Melbourne; John Didlick, Hepatitis Australia; John G and Jess Doumany, Australian Injecting and Illicit Drug Users League; Lisa Bastian, Western Australia Department of Health; Mandy Charleton, Ministry of Health; Manoji Gunathilake, Royal Darwin Hospital; Megan Campbell, National Aboriginal Community Controlled Health Organisation; Mish Pony, Scarlet Alliance; Norm Roth, Prahran Market Clinic; Philip Cunningham, SydPath; Phillip Read, Kirkton Road Centre; Rick Varma, Sydney Sexual Health Centre; Tom Rees, South Australia Ministry of Health; and Thi Nguyen, Victoria Polkinghorne, Michael Traeger, and Nyssa Watson, Burnet Institute. The authors also acknowledge all clinics participating in ACCESS, including the site investigators, Dr Matthew Penn, who contributed data to this analysis. ACCESS is a collaboration between the Burnet Institute, Kirby Institute, and National Reference Laboratory.

***Data availability*.** Data are not publicly available.

***Financial support*.** ACCESS receives core funding from the Australian Department of Health with the aim to monitor Australia's progress in the control of blood-borne viruses and sexually transmitted infections. In addition, the governments of New South Wales, Victoria, and Western Australia provide funding for state-level outcomes. Funding for particular outcomes is also provided by the BBV & STI Research, Intervention and Strategic Evaluation Program (BRISE); a National Health and Medical Research Council (NHMRC) Project Grant (grant number APP1082336); an NHMRC Partnership Grant (grant number GNT1092852); and the Prevention Research Support Program, funded by the New South Wales Ministry of Health.
